# Implementation of a Brief Dialectical Behavioral Therapy Skills Group in High Schools for At-Risk Youth: Protocol for a Mixed Methods Study

**DOI:** 10.2196/32490

**Published:** 2022-05-12

**Authors:** Tamika Zapolski, MacKenzie Whitener, Shirin Khazvand, Queenisha Crichlow, Rebecca Revilla, Eduardo F Salgado, Matthew Aalsma, Melissa Cyders, Michelle Salyers, Wei Wu

**Affiliations:** 1 Department of Psychology School of Science Indiana University–Purdue University Indianapolis Indianapolis, IN United States; 2 University of Alabama at Birmingham Birmingham, AL United States; 3 University of Alabama Tuscaloosa, AL United States; 4 Department of Pediatrics School of Medicine Indiana University Indianapolis, IN United States

**Keywords:** dialectical behavioral therapy, adolescents, high school, intervention, high school, teenagers, risk-taking behavior, impulsivity, emotion dysregulation, social and emotional learning, youth

## Abstract

**Background:**

Adolescence is a developmental period marked by engagement in risk-taking behaviors, especially among impulsive or emotionally dysregulated youth. Thus, interventions that teach skills to reduce the risk of negative outcomes associated with emotional dysregulation are required. Social and emotional learning (SEL) programs have been developed to address both adolescent emotional dysregulation and risk-taking behaviors; however, current programs have mostly been implemented among younger youth and are used as a tier 1 universal intervention rather than a targeted tier 2 intervention for youth identified with emotional regulation difficulties.

**Objective:**

This study aimed to address the need for SEL programming that can be delivered in schools, particularly for older youth who have difficulties with emotional or behavioral dysregulation, to reduce the risk of health-risk behaviors among this population.

**Methods:**

Here, we outline the implementation of an SEL intervention titled *Going 4 Goals*, a 9-session adaptation of the Dialectical Behavioral Therapy for Adolescents (DBT-A) program delivered to at-risk high school students in a school setting. The primary objectives of the study are to test whether participating in the skills group intervention produces significant increases in the core DBT-A skills of mindfulness, emotional regulation, distress tolerance, and interpersonal effectiveness, while also producing significant decreases in substance use and risky behaviors. These primary outcomes are based on changes in participant scores between baseline and after the intervention and follow-ups at 1, 3, and 6 months compared with a control group of youth participating in the school’s health curriculum at the same time points. Qualitative interviews will also be conducted with intervention participants and school staff to examine acceptability and facilitators of and barriers to the intervention.

**Results:**

A total of 171 participants across 13 groups had been enrolled in the intervention, with data collection ending December 2021. Data analysis will begin in the spring of 2022, with expected results to be published in the spring of 2023.

**Conclusions:**

This paper describes the protocol of the 9-session school-based adaptation of the DBT-A intervention and discusses the strengths and limitations of the study and future directions.

**International Registered Report Identifier (IRRID):**

DERR1-10.2196/32490

## Introduction

### Background

Adolescence is a developmental period characterized by an increase in risk-taking behavior [1-3] manifested in several areas, including reckless driving, unprotected sexual behavior, and substance use [4]. Although an increase in some risk-taking behaviors is common among adolescents, addressing engagement in such behaviors is warranted because they are associated with the leading causes of death among adolescents (eg, injury deaths from motor vehicle crashes, firearms, and suffocation) [5]. In addition, adolescents who experience higher emotional dysregulation and impulsivity are especially vulnerable to the negative health outcomes of risky behaviors [6,7].

Social and emotional learning (SEL) programs have been developed to primarily address adolescent emotional dysregulation and risk-taking behaviors within school settings [8,9]. The term SEL was first coined in 1994 when the Collaborative for Academic, Social, and Emotional Learning (CASEL) was founded [10]. Through SEL programming, youth can acquire and effectively apply the knowledge, attitudes, and skills necessary to understand and manage their emotions, establish and achieve positive goals, develop and maintain positive relationships, and make healthy and responsible decisions [10]. In turn, this knowledge and skill development aids in youth’s ability to attain and maintain personal well-being across their life span [10]. Thus, the core components of SEL programs that align with the five competencies identified by the CASEL focus on improving (1) self-awareness of one’s emotions, thoughts, and behaviors; (2) self-management to regulate one’s emotions, thoughts, and behaviors effectively; (3) social awareness and skills; (4) relationship skills to form and maintain healthy relationships; and (5) responsible decision-making to make constructive and respectful choices [11-13].

SEL programs have been associated with multiple positive outcomes, including increases in social skills and prosocial behaviors and decreases in antisocial and externalizing behaviors among youth [14-17]. For example, a meta-analysis of postintervention and long-term outcomes of school-based SEL programs among youth in elementary, middle, and high schools found that participants who received the intervention had better outcomes than those in the control group at postintervention on social-emotional skills, social-emotional attitudes toward self, others, and school, emotional distress, academic performance, and drug use (effect size=0.12-0.22). These improvements were also found in follow-up assessments occurring between 6 months and 18 years after the intervention for all outcomes as well as prosocial behaviors and conduct disorder (effect size=0.13-0.33) [17].

Despite the clear benefits of SEL programs on health outcomes for youth, there are gaps in the generalizability of the findings. First, research on the implementation of SEL programs has been primarily conducted among younger youth (eg, in elementary or middle school) [16], with limited research testing the efficacy of SEL programs among older youth in high school settings [18]. For example, of the 213 studies included in a meta-analysis conducted by Durlak et al [16], only 13% were from high school settings, with a similar percentage of studies found in the meta-analysis conducted by Taylor et al [17]. Second, there is limited empirical evidence on the efficacy of SEL programs as targeted interventions (also referred to as *tier 2* interventions) for youth experiencing social, emotional, or behavioral problems and at increased risk of negative health outcomes [19]. For example, Blewitt et al conducted a systematic review of targeted tier 2 SEL interventions and found that only 19 studies met the inclusion criteria. However, the findings indicated positive outcomes, with the tier 2 SEL programs strengthening participants’ social and behavioral functioning [19]. Yet, the findings are limited in generalizability in that the review focused only on tier 2 SEL programs delivered in early childhood education and care settings, with much of the evidence directed primarily toward preschoolers with externalizing problems. Thus, there is a gap in the literature regarding the efficacy of tier 2 SEL interventions for older at-risk adolescents.

Dialectical behavioral therapy (DBT) is an effective tier 2 SEL intervention for social, emotional, and behavioral problems among youth in high schools. DBT was originally developed by Linehan et al [20] to treat chronically suicidal adults, many of whom were diagnosed with borderline personality disorder [20]. DBT uses cognitive behavioral and mindfulness techniques to address difficulties in four specific areas—distress tolerance, mindfulness, emotional regulation, and interpersonal effectiveness—and has been proven to be effective in treating many mental disorders [20-22]. Owing to the success of the intervention among adults, adaptations of DBT have been created for adolescents, particularly those with similar clinical symptoms or difficulties with emotional dysregulation [23,24]. The format of both DBT and Dialectical Behavioral Therapy for Adolescents (DBT-A) consists of individual therapy and a skills group component; however, DBT-A differs from DBT in that some content is adapted to be developmentally appropriate for youth, and the length of treatment is reduced from 1 year to 24 weeks [23,24]. Outcome data for DBT-A have demonstrated the effectiveness of the intervention in treating various mental and behavioral health conditions in adolescents, including suicidality, emotional dysregulation, depression, and anger across multiple clinical settings, including correctional facilities, residential in-patient programs, and day treatment programs [23,25,26].

DBT-A has also been adapted for implementation in nonclinical settings, such as schools, with the publication of the DBT Skills in Schools: Skills Training for Emotional Problem Solving for Adolescents (DBT STEPS-A) manual [27,28]. In the DBT STEPS-A manual, the authors positioned DBT as an SEL curriculum given its focus on understanding and managing emotions, developing and maintaining relationships, and responsible decision-making, which align with the CASEL principles [27,28]. A critical limitation, as also noted by other scholars, is that of the many existing SEL interventions, there is a lack of explicit attention to emotional processing, such as learning how to cope, regulate emotions, or modify factors causing emotional distress [29], which is a key component of DBT interventions. In addition, the authors of the DBT STEPS-A manual discuss the use of the program as both a universal tier 1intervention—for all students, delivered within a classroom curriculum—and a targeted tier 2 intervention for students who need additional support for their social and emotional needs delivered in small groups [27,28].

However, few studies have examined the efficacy of DBT-A or DBT STEPS-A in school settings or as targeted tier 2 interventions. To our knowledge, only 6 studies have been published, 5 of which were conducted among high school youth. It is also important to note that 3 of the studies were conducted before the publication of the DBT STEPS-A and were therefore conducted using an adaptation of the DBT or DBT-A protocol. The remaining 4 studies used the DBT STEPS-A manual. Notably, all studies included only the group skills component and modified the length of treatment to 6 to 22 sessions across 4 to 22 weeks.

The first study was conducted by Richard et al [30], who adapted the original DBT skills group protocol created by Linehan et al [20] to address the behavioral distress needs of youth in a disciplinary alternative education program. The protocol included 8 to 10 group sessions lasting 40 to 45 minutes, which occurred twice a week for 4 weeks. Their study included 125 students aged 6 to 18 years who primarily identified as Hispanic. The findings indicated that participation in the group was associated with reductions in behavioral distress compared with youth who did not receive the intervention [30]. A second study by Zapolski and Smith [31] also found promising results for at-risk middle school youth. Similar to the Ricard et al study [30], Zapolski and Smith adapted the original DBT skills group for adults by Linehan et al [20] to a 9-session skills group protocol for middle school youth. Among the 53 students (most in seventh grade, mean age 12.7 years who participated in the group, the findings indicated that the intervention effectively decreased self-reported engagement in risky health behaviors and intentions to engage in risky behaviors. Moreover, these findings were more pronounced among youth who reported higher impulsivity scores [31].

A third study published by Flynn et al [32] differs from the first two in that it was conducted outside the United States, in Ireland, and used the DBT STEPS-A manual [27]. Moreover, the researchers adapted the DBT-A program to be delivered across 22 weeks rather than 30 weeks, as originally proposed by Mazza et al [27] Positive outcomes were found, such that among their sample of 72 girls aged 15 to 16 years, participation in the group intervention was associated with significant improvements in emotional distress symptoms and internalizing problems compared with a control group of youth who did not receive the intervention [32]. The fourth study, by Martinez et al [33], also implemented the DBT STEPS-A manual [27] and was conducted with 42 ninth grade students enrolled in a rural southeastern high school in the United States. The program consisted of 20 sessions delivered across 12 weeks, and the findings indicated a treatment effect. Participants in the intervention reported significant improvements in social resiliency and difficulties with emotional regulation compared with youth in the control group (ie, required health or physical education course). The fifth study, which was also conducted in the United States but within low-income schools, was published by Chugani et al [29]. The program was adapted to 19 sessions delivered primarily once a week in high schools within a large northeastern school district. Although the study did not focus on student outcomes, the findings indicated high acceptability and feasibility among teachers conducting the intervention in schools within the health curriculum.

Although promising data exist based on the studies cited, there have also been mixed findings. Burckhardt et al [34] adapted the original DBT protocol for adults [20] to a 6-session skills group, with each session lasting 50 minutes. The results indicated, among their sample of 50 youth aged 14 to 16 years, that participation in the intervention was associated with small *increases* in anger, symptoms of anxiety, and depression based on both the postintervention and 6-month follow-up assessments. The researchers hypothesized that this finding could be related to the focus on mindfulness in DBT and the ability to *open up*, which may result in greater awareness, and thus the reporting of symptoms of anxiety and depression. This study also indicated that the control group, which consisted of youth who attended usual classes that involved learning material regarding future careers, had better emotional regulation scores than the intervention group [34]. However, some positive findings were observed for the intervention group based on qualitative interviews conducted among participants—74% of them reported positive benefits of the intervention, including being able to better regulate their emotions.

In conclusion, despite the benefits of SEL programs for adolescents in reducing the risk of engagement in risk-taking health behaviors, much of the existing empirical support for SEL programs is based on evidence from younger youth and has rarely been tested as a tier 2 intervention targeting youth at greater risk of experiencing adverse health outcomes. Using DBT-A can address these gaps by providing an evidence-based intervention that shows promise in addressing risk-taking behavior among older at-risk youth in school settings. However, research implementing DBT-A in schools for older youth, particularly as a tier 2 intervention, is limited, and more empirical support on its efficacy is needed.

### Objectives

In this study, we aim to fill this critically important research and clinical gap by implementing a 9-session adaptation of the DBT-A [24] and DBT STEPS-A [27] manual, titled *Going 4 Goals*, in 2 public high schools for at-risk youth identified by school staff. The primary objectives of the study are to test whether participating in the skills group intervention produces significant increases in core DBT-A skills (ie, mindfulness, emotional regulation, distress tolerance, and interpersonal effectiveness) and significant decreases in substance use and risky behaviors. These outcomes are based on changes in participant scores between baseline and postintervention assessments and 1-, 3-, and 6-month follow-ups compared with a control group of youth participating in the school’s health curriculum at the same time points. A secondary objective is to examine the acceptability, facilitators, and barriers of the intervention through qualitative interviews with intervention participants and school staff. This paper describes the protocol of the 9-session school-based adaptation of the DBT-A intervention.

## Methods

### Study Design Overview

**Figure 1:**
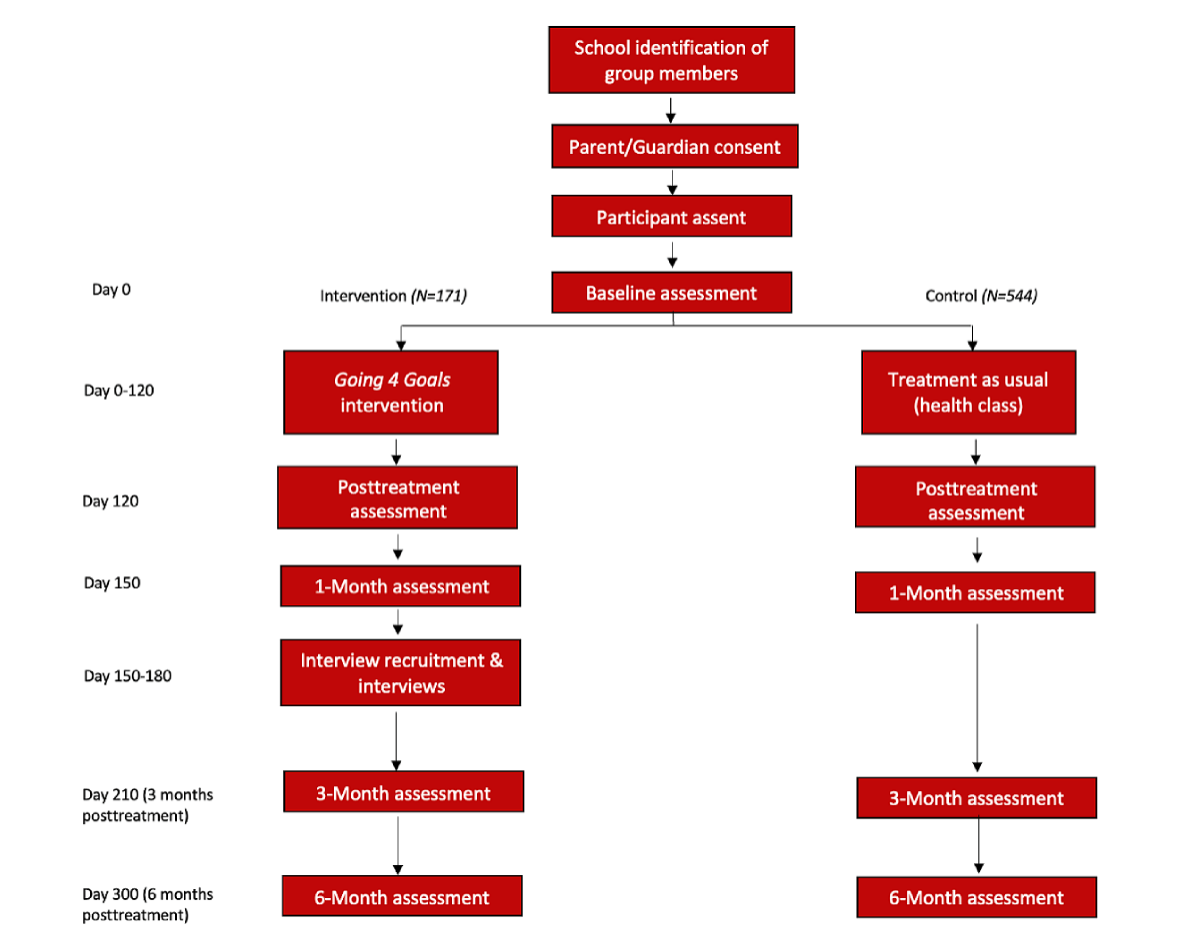
Study flow.

A mixed methods design was adopted to explore the implementation and efficacy of the school-based DBT-A skills group, titled *Going 4 Goals*, for at-risk high school youth. Youth are identified by school staff to participate in the skills group, which is being held during school hours at local high schools and occurs during noncore instructional class periods. An opt-out consent process for guardians and an active assent procedure for youth is being used, with all eligible participants included in the intervention groups. Control participants are also being enrolled to compare outcomes. Control group students are from health classes at the school where the intervention is taking place. All participants (intervention and control) will take a quantitative survey at 5 time points (baseline; after the intervention, ie, approximately 9 weeks after baseline; and 1, 3, and 6 months after the intervention) on paper or electronically through Qualtrics, a secure research survey software provided through the university. The survey includes measures to evaluate the core skills of the DBT-A program: emotional dysregulation, distress tolerance, mindfulness, and interpersonal effectiveness and other key study variables, including impulsivity, substance use, and risk-taking behaviors. At the completion of each 9-session intervention program, participants and school staff will be asked to participate in a qualitative interview approximately 1 month postintervention to understand the facilitators and barriers to the program. The study was approved by the Indiana University–Purdue University Indianapolis institutional review board (#1610685795) on July 25, 2018. Data collection began in July 2018 and ended December in 2021. Figure 1 illustrates the implementation timeline. Details regarding the methodology of the study protocol are provided in the subsequent sections.

### Study Recruitment

The principal investigator and project manager reached out to several schools in the Indianapolis area to inquire about their interest in partnering to implement the *Going 4 Goals* program with their students. Two schools agreed to implement the intervention during school days. Both schools are diverse, with high rates of free or discounted lunch recipients (76% and 50.1%, respectively) and median household incomes of US $49,175 and US $62,829, respectively. Both schools are also diverse in relation to race and ethnicity, with approximately 70% of the students at both schools identifying as non-White. Intervention participants are identified by the school staff based on who they deem as either *at risk* (eg, prior school-related drug offense, conduct problems, engagement in risky health behaviors or school fights, or had in-school or out-of-school suspension) or believe could benefit from learning the core DBT-A skills (ie, mindfulness, emotional regulation, distress tolerance, and interpersonal effectiveness). The most common identification measures used by schools are (1) teacher recommendation based on class attendance and student behavior in class and (2) guidance counselor recommendation based on student behavior referral records (ie, number of detentions, suspensions, or expulsions on students’ records), attendance records, or known substance use.

### Consent and Assent

Parent or guardian approval is being used based on an opt-out parent or guardian consent process. A letter is sent to the identified youth’s legal guardian on behalf of the school and the research team, indicating that their child has the option to participate in a group that aims to reduce the risk of substance use and other risky health behaviors by teaching skills to help manage emotions, stress, and interpersonal conflicts. The letter describing the study’s purpose, risks, benefits, and inclusion and exclusion criteria (ie, students are enrolled in the school; are able to adequately speak, understand, and read English; and are not concurrently receiving mental health services for the duration of the intervention program) is sent to the guardian through the US mail or sent home by the school administration with each student. The legal guardian is asked to sign and return to the bottom of the letter if they do not want their child to participate in the intervention. Guardians are given 2 weeks to return the opt-out consent form. After the 2-week period for guardians to return consent forms has passed, all youth who are still eligible are asked to attend an information session regarding the intervention group during school hours. A brief overview of the intervention is provided, and the youth who wish to participate sign assent forms and complete a survey assessing baseline measurements of the study variables. Participants are also informed about the opportunity to complete a qualitative interview after completing the skills group to understand the facilitators and barriers of implementation. For participants who wish to participate in the interviews, a new assent form is provided and signed by them at the end of the intervention to record and transcribe the interviews.

Control participants complete a similar consent and assent process. A consent form is sent home to their parent or guardian with a 2-week window to return it to the school before the youth can assent to participate in the study. Those youth who consent are then asked to complete the survey at school during their health class.

### Ethical Approval and Consent to Participate

All procedures performed in this study involving human participants were in accordance with the ethical standards of the institutional and/or national research committee and the 1964 Helsinki declaration and its later amendments or comparable ethical standards. The study protocol was approved by the Indiana University–Purdue University Indianapolis institutional review board (#1610685795). Opt-out consent for participation will be obtained from the youth's legal guardian. Written voluntary informed consent will be obtained from all youth participants. Confidentiality will be maintained, except if participants are at risk of significant harm or request assistance.

### Intervention Procedures

#### Going 4 Goals

Youth who participate in the *Going 4 Goals* group intervention will attend 9 sessions conducted once a week during school hours, lasting approximately 40 minutes (ie, 1 class period). The intervention sessions were first taken from the DBT-A manual [24], which provided 2 sessions for the mindfulness and 4 sessions for the emotional regulation, distress tolerance, and interpersonal effectiveness modules. To reduce the material to fit within a 9-session intervention, the principal investigator of the study consulted with other clinical psychologists trained in DBT to identify the primary skills related to the 4 DBT components that could be delivered within nine 40-minute sessions. The selected skills and text discussing them were then taken directly from the DBT STEPS-A manual [27] to align with how the skills would be presented to students within a nonclinical school setting. To this end, of the 9 sessions, the first was devoted to orientating the students to the intervention, goal setting, and introduction to mindfulness, the second session was devoted to mindfulness skills, the third through fifth sessions were devoted to emotional regulation skills, the sixth and seventh sessions were devoted to distress tolerance skills, and the eighth session was devoted to interpersonal effectiveness skills. The ninth session reviewed all skills learned in previous sessions and included the postintervention assessment. Each session begins with a mindfulness exercise and a didactic period in which skills related to emotional regulation, distress tolerance, interpersonal effectiveness, and mindfulness taken directly from the DBT STEPS-A manual are taught. There is also time incorporated within each session for active youth participation, with at-home practice assigned at the end of each session. See Textbox 1 for a complete overview of the sessions and objectives.

***Going 4 Goals* session overview.**

**Sessions and their objectives**

- Session 1: Introduction
  Give students an overview of the program and its purpose.
  Present the rewards system to the students for attainment of each goal.
  Complete pretreatment survey.
  Mindfulness introduction.
- Session 2: Mindfulness skills
  Teach students how to be aware of their emotions without necessarily changing them.
- Session 3: Understanding emotions
  Teach students how to observe and describe emotions.
  Help students understand the function of emotions.
- Session 4: Reducing vulnerability to extreme emotions.
  Teach students the importance of taking care of their body and its influence on emotional reactivity (ie, balanced eating, adequate sleep, exercise, etc).
- Session 5: Managing emotions or opposite action
  Teach students how to experience emotions without immediate mood-based action.
  Teach students how to change or reduce the intensity of their emotions through opposite action.
- Session 6: Distress tolerance or relaxation
  Teach students strategies to help manage mood during particularly difficult emotional periods (eg, getting bad grade).
  Teach relaxation training to students to help reduce intense negative emotions.
- Session 7: Perspective taking, problem solving, and pros and cons
  Teach students how to obtain a more objective assessment of distressing situations.
- Session 8: Relating to others
  Thinking mistakes.
  Discuss validation of others and self-validation.
  Teach students how to best communicate with others, so that can either: (1) maintain relationships and reduce conflict, (2) get what they want or say no, or (3) keep their self-respect.
- Session 9: Review of skills
  Review skills taught over the course of the program with application exercise.
  Complete posttreatment survey.

#### Control: Treatment as Usual

Participants assigned to the control group or treatment as usual are all enrolled in a health education class at their school, which is a state requirement for all high school students. These students will not receive any *Going 4 Goals* programming.

### Data Collection Procedures

For the intervention participants, quantitative surveys are completed during the first meeting of the *Going 4 Goals* program as a baseline measure. The surveys are completed again at the end of the ninth session and at 1-, 3-, and 6-month follow-ups. In addition, during the first group session, each participant will set both an academic and personal goal with 3 smaller tasks to reach the goal using the SMART (ie, specific, measurable, attractive, realistic, and timely) framework [35]. These goals are later revisited in sessions 5 and 9, where progress is self-reported. For control participants, the same quantitative surveys are collected in the health education class in the same school that the intervention participants attend using the same timeline (at baseline; approximately 9 weeks after the baseline assessment; and 1-, 3-, and 6-month follow-ups). Control participants will not make SMART goals as part of this study. A snack and pen are given to each participant as an incentive for completing the baseline and posttreatment surveys. For the follow-up surveys, all participants will receive monetary compensation in the form of gift cards for each completed survey (US $10 for 1 month, US $15 for 3 months, and US $20 for 6 months of follow-up).

Qualitative interviews are also conducted with the intervention participants and school staff either in-person or on the phone approximately 1 month after the intervention to assess the impact of the program, facilitators, and barriers to implementing the intervention. Interviews will be conducted by trained research staff, with steps taken to reduce the likelihood that the group leaders are conducting interviews with students in their own groups. The interviews will last approximately 30 minutes. Youth participants will receive a US $10 gift card for completing the interview, and the school staff will receive a US $25 gift card.

### Measures

#### Quantitative Measures

##### Demographics

Demographic information is collected during each data collection, starting at baseline. Participants are asked to report their age, gender identity, race, ethnicity, primary spoken language, grade in school, and mental health diagnoses (if any).

##### Emotion Dysregulation

The Emotion Dysregulation Scale short version [36] is a 12-item instrument used to examine emotional experience, cognition, and behavior. The scale consists of items scored on a 7-point Likert scale ranging from 1 (*not true*) to 7 (*very true*) specific to each aspect of emotional experience (eg, “Emotions overwhelm me”), cognition (eg, “When I’m upset, everything feels like a disaster or crisis”), and behavior (eg, “When my emotions are strong, I often make bad decisions”). The internal consistency has been shown to be high for each subscale (Cronbach α=.93 to .95 [36]).

##### Impulsivity

The Urgency, Premeditation, Perseverance, Sensation Seeking, Positive Urgency, Impulsive Behavior Scale modified for children [37] is used to assess impulsivity with five 8-item subscales measuring separate impulsivity-related traits: negative urgency, positive urgency, lack of perseverance, lack of premeditation, and sensation seeking. Example items of each scale include negative urgency (eg, “If I feel like doing something, I tend to do it, even if it’s bad”), positive urgency (eg, “When I am in a great mood, I tend to do things that could cause me problems”), lack of perseverance (eg, “I finish what I start”), lack of premeditation (eg, “I tend to stop and think before doing things”), and sensation seeking (eg, “I like new, thrilling things to happen”). Participants responded to items on each subscale on a 4-point Likert scale, 1 (*not at all like me*), 2 (*not like me*), 3 (*somewhat like me*), and 4 (*very much like me*), with items coded so that higher scores indicate more impulsive tendencies. Internal consistency has been shown to be high in previous research among youth (Cronbach α=.81 to .90 [37]).

##### Substance Use

The substance use history measure was adapted from various national studies conducted among youth (eg, Monitoring the Future and Youth Risk Behavior Surveillance System). It consists of 9 items and evaluates substance use in the past 30 days. Participants are asked to indicate how many days they used a substance in the previous month (0, 1-2, 3-5, 6-9, 10-19, 20-29, every day). The substances evaluated are cigarettes (2 items: smoked at all and smoked half a pack or more), smokeless tobacco, alcohol (2 items: had at least 1 drink and had 5 or more drinks in a row), cannabis, inhalants, other drugs (eg, lysergic acid, cocaine, and methylenedioxymethamphetamine), and e-cigarettes.

##### Distress Tolerance

The Distress Tolerance Scale [38] consists of 15 items that measure self-evaluations and expectations of experiencing negative emotional states. Example items include “My feelings of distress are so intense that they completely take over” and “I’ll do anything to avoid feeling distressed or upset.” Items are rated on a 5-point scale, 5 (*strongly disagree*), 4 (*mildly disagree*), 3 (*agree and disagree equally*), 2 (*mildly agree*), and 1 (*strongly agree*), with higher scores indicating higher distress tolerance. The Distress Tolerance Scale has been demonstrated to have high internal consistency (Cronbach α=.89 [38]).

##### Mindfulness

The Philadelphia Mindfulness Scale (PHLMS [39]) is used to measure key constituents of mindfulness: present-moment awareness (eg, “I am aware of what thoughts are passing through my mind”) and acceptance (eg, “There are aspects of myself I don’t want to think about”). It comprises 20 items rated on a 5-point scale—5 (*very often*), 4 (*often*), 3 (*sometimes*), 2 (*rarely*), and 1 (*never*). Higher scores on the *PHLMS* *awareness* subscale are associated with higher mindful attention and awareness, whereas higher scores on the *PHLMS acceptance* subscale are associated with less thought suppression and rumination. The PHLMS has shown good internal consistency across clinical and nonclinical samples (Cronbach α=.075 to .91 [39]).

##### Interpersonal Effectiveness

The Peer Conflict Scale-Youth [40] is used as a proxy for interpersonal effectiveness, as it assesses reactive and proactive aggression. The measure consists of 20 items: 10 items examining proactive aggression, both proactive overt items (eg, “I start fights to get what I want”) and proactive relational items (eg, “I gossip about others to become popular”), and 10 items examining reactive overt (eg, “When someone hurts me, I end up getting into a fight”) and relational aggression (eg, “If others make me mad, I tell their secrets”). Items are rated on a 4-point Likert scale—0 (*not at all true*), 1 (*somewhat true*), 2 (*very true*), and 3 (*definitely true*). Previous studies have established a high internal consistency (Cronbach α=.93) [41].

##### Risky Behaviors

The Mood-Based Questionnaire-Children [37] is a self-report measure that assesses lifetime endorsement and the current likelihood of engaging in 24 risky behaviors while being in either an unusually negative mood or an unusually positive mood. Lifetime endorsement is measured on a dichotomous yes or no scale. The likelihood of engaging in risky behaviors is measured on a 5-point Likert scale, with 1 (*not at all*), 3 (*maybe*), and 5 (*will definitely try*) points. Behaviors assessed on the measure include drinking alcohol, breaking the law, smoking a cigarette or cigar, kissing someone romantically, urinating outside, shoplifting, starting a fight, trespassing, cheating on a test, and disobeying parents. In previous research on adolescents, there has been good evidence of the reliability of the Mood-Based Questionnaire-Children (Cronbach α=.85 to .92 [37]). In this study, 3 modifications were made. First, the mood component was removed from the instructions; thus, the measure assesses the likelihood of engaging in risky behavior regardless of mood state. Second, the timeframe was modified to assess risk taking within the past month rather than lifetime endorsement. Third, an item not included in the original measure was added to assess any cannabis or marijuana use.

##### SMART Goal Tracking

One of the components of *Going 4 Goals* is creating and tracking SMART goals. Intervention participants are taught that SMART goals should be specific, measurable, attractive, realistic, and timely. Group leaders will assist participants in creating a personal and an academic SMART goal to work on throughout their time in the program, each with 3 smaller tasks that will help them reach their overall goal. Participants then rate themselves on a scale of 1 to 10, indicating their progress toward reaching their goals, with 10 indicating goal attainment. Participants will then re-evaluate their progress in sessions 5 and 9. These goals will not be shared with other participants. Control participants will not set any SMART goals for this study.

#### Qualitative Measures

Semistructured interviews are conducted with intervention participants and school staff to understand factors related to implementation and program outcomes, including appropriateness for a school-based setting; acceptability by participants; feedback on the logistics and makeup of the group; opinions about group topics, group leaders, and style of delivery; and influence on mental, behavioral health, educational, and social outcomes (Textbox 2). Interviews will be appropriately tailored for each type of interviewee, audiotaped, and last approximately 30 minutes.

**Qualitative interview questions.**

**Categories and example questions**

- *Going 4 Goals* Impact
*How did Going 4 Goals impact your day-to-day life?*
*How did it impact your relationships parents, peers, and teachers?*
- Process
*Tell me about any skills you may have used from the group.*
*What did you like most or least about (skills)?*
*What were the most or least helpful group activities you participated in?*
- Design
*Have you participated in any other groups related to stress management?*
*If so, how did it compare to Going 4 Goals?*

### Confidentiality

To protect confidentiality, each participant is assigned a subject ID number connected only to their name on a file stored on a secured network and server maintained by the research staff behind a university firewall. The ID number is used for all data collection components (questionnaires, qualitative transcriptions, and goal sheets). All completed informed consent and assent documents are stored in a locked file cabinet inside a locked office. All electronic data (quantitative data files, audio files, and qualitative transcriptions) are also stored on a secured network and server maintained by the research staff behind a university firewall. Contact sheets with participants’ email, phone numbers, and addresses, which are used for follow-up interviews and surveys, are stored in a separate locked cabinet inside a locked room. Contact information collected electronically is stored behind a university firewall on a secure, password-protected, restricted-access server.

Intervention participants and their guardians are also assured that student discussions in the *Going 4 Goals* sessions, responses to surveys, and information given during interviews will not be shared with anyone outside of the research team, except for specific circumstances in which the research team needs to breach confidentiality (eg, reports of suicidal or homicidal ideation and child abuse or neglect). Thus, in most circumstances, parents, teachers, or school administrators will not have access to individual responses to the study. When the study results are shared with school administrators, no participant names or ID numbers are included in the aggregate data.

### Data Analytic Plan

For the quantitative data, we plan to conduct linear mixed models to examine whether there are significant changes in emotional regulation, distress tolerance, mindfulness, and interpersonal effectiveness skills at the postintervention assessment compared with baseline in the intervention group. Linear mixed models will also be used to examine significant changes in past 30-day substance use and the likelihood of engaging in substance use and risky health behaviors at the postintervention assessment compared with baseline assessment. We will compare changes in these outcome measures with those of youth in the control group. In addition, as some students may not be present in all sessions, we will test whether there is evidence of a relationship between the number of sessions attended and the outcomes.

For the qualitative data, after interviews are completed, qualitative audio files will be compiled and sent to an external company for transcription. A coding team of 4 research assistants and a project manager will review all transcribed interviews to create coding categories for each question. Student interviews will be split into 2 sections (*impact and skill use* and *logistics*), and administrator and teacher interviews will be coded whole. The team uses Atlas.ti (ATLAS.ti Scientific Software Development GmbH) to create qualitative tables and manually review each transcript to pull relevant quotations from each interview. All coding is also reviewed by another team member for reliability. Finally, qualitative summaries will be created by the coding team and placed in quantitative tables for dissemination.

## Results

A university-supported initiative provided funding for this study. Data collection began in July 2018 and was completed December 2021. Participant recruitment began in August 2018*.* Thirteen groups have been implemented, with 171 participants enrolled in the intervention. In addition, of the 171 participants enrolled in the *Going 4 Goals* program, 146 (85.4%) completed the program, resulting in a retention rate of 85.4%.

The youth participating in the intervention were primarily in the ninth grade (age range 12-16, mean age 14.3 years; SD 3.1 years). The school staff targeted ninth graders to participate in *Going 4 Goals* because of the limited number of mental health and SEL services provided for this age group and difficulties school staff have witnessed with students transitioning to ninth grade (ie, transitioning from middle school to high school amid puberty). Of the 171 youth enrolled in *Going 4 Goals*, most were male (124/171, 72.5%) but diverse in terms of race and ethnicity (61/171, 35.7% Black; 55/171, 32.2% Hispanic or Latino; 42/171, 24.6% White; 3/171, 1.7% Native American or American Indian; and 18/171, 10.5% multiracial). The control group was equally divided by gender (49.4% male), with race and ethnicity mirroring school demographics. Finally, 32.2% (55/171) of the intervention group reported having a mental health diagnosis, compared with 71.3% (122/171) of the control group. Qualitative interviews have also been conducted. To date, 36 students, 7 teachers, and 4 school administrators have completed qualitative interviews. This study is expected to conclude in December 2022.

## Discussion

### Principal Findings

This paper outlines the background, research design, and intervention components of *Going 4 Goals*, a 9 session DBT-A skill group intervention for at-risk youth implemented in high schools. The proposed study is novel in that there is limited empirical evidence available in the literature on the efficacy of tier 2 SEL interventions for at-risk high school youth, which is critical as such youth are at greater risk for engagement in health-risk behaviors. Thus, receiving appropriate evidence-based services that can reduce such risk is needed. This study aims to fill this important research and clinical knowledge gap.

Implementing the *Going 4 Goals* intervention in high school youth has additional strengths. First, implementing *Going 4 Goals* in schools increases access to mental health services for at-risk youth who may otherwise not have access to such resources. Implementing this program in schools also makes it easier to engage and maintain communication with participants, which can help keep youth engaged and active during the intervention. As mentioned, the participant retention rate has been high at 85.4%. Second, we were able to partner with engaged school administrators who saw the benefits of the program and understood how participants could also enhance their academic performance. Thus, the intervention group is being implemented during the students’ homeroom class period. This is done so that there would be minimal disruption to the students’ schedules and to ensure that they are still attending their core classes and not missing any foundational curriculum. Holding groups during homeroom also protects teachers’ instruction time, minimizing the disruption in scheduling for both teachers and students. Third, given the minimal number of materials needed to implement the program, this is a low-cost intervention, meaning that it can easily be implemented across different school systems and sustained in the long term. Finally, our intervention does not require facilitators to hold special certifications or advanced degrees to deliver the content, meaning that teachers, administrators, and other school staff can administer *Going 4 Goals*. In this study, graduate students serve as group facilitators, with undergraduate students helping as cofacilitators. These students do not have any special certifications or are required to have any specific training other than being trained in the *Going 4 Goals* or DBT-A protocol to lead the groups. A future direction of our work is to train school staff to deliver the intervention, which will establish the feasibility of implementing the intervention by nonresearchers or clinicians.

In addition to the strengths of this intervention, there are also some challenges and lessons learned for future research in this area. First, as we began implementing the intervention, we had some issues with students’ punctuality and remembering to attend regularly. To mitigate this, we found that sending reminder text messages the morning of the group, delivering a school-wide announcement over the intercom, and having homeroom teachers remind students individually were effective ways to increase attendance rates. Email reminders were also sent to students but were not well-received in the age group we serviced. Specifically, students indicated that they do not regularly check their email and thus did not find the email reminders beneficial. Second, scheduling recurring weekly sessions was challenging due to school breaks, state testing, weather delays, field trips, and other unforeseen circumstances. This would, at times, alter our schedule; thus, emphasis on constant communication with schools is crucial. We found that obtaining a finalized semester calendar from the school ahead of time was helpful when working on logistics for the group.

Furthermore, because our research team provides the intervention groups and can only attend at a specified time 1 day per week, any changes or cancelations related to school events meant that we had to completely cancel the session for the week. However, if school staff are trained as facilitators, there will be more flexibility in implementing the intervention, and last-minute school schedule changes will not cause major disruptions in the timeline of the group. Training school staff facilitators will also aid in increasing the access and reach of the intervention to more students and address issues of long-term sustainability in school systems. Third, we found that hosting the groups during school days can be difficult because a designated room is required for the group to meet each week. This issue can also be addressed by training school staff, as they can use their own classrooms or office space for group sessions and have more flexibility in the time of day to hold groups based on room availability. Also related to scheduling was the class period during which the group was held. Generally, groups are conducted during a homeroom or study hall class period to avoid interference with core coursework; however, this may be problematic for schools that do not have a homeroom or study hall period built into their schedules. Therefore, alternative ways to fit the program into school hours may be challenging in other settings.

### Future Directions and Dissemination Plans

We anticipate that *Going 4 Goals* will be effective in equipping at-risk students with skills to better cope with stress and reduce their engagement in risky behaviors, such as substance use. Through the completion of qualitative interviews with group participants and school administrators, we will also gain important information on the facilitators, barriers, and attitudinal drivers to enhance tier 2 SEL interventions in high school settings. Such findings will help glean valuable information regarding how best to implement tier 2 SEL and mental health programming to reduce risk taking and increase emotional regulation among at-risk adolescents in high school settings. Future plans include developing procedures to sustain the intervention in schools by training school staff in the intervention protocol and expanding the implementation of the program to other school systems.

## Abbreviations

CASEL: Collaborative for Academic, Social, and Emotional Learning
DBT: dialectical behavioral therapy
DBT-A: Dialectical Behavioral Therapy for Adolescents
DBT STEPS-A: DBT Skills in Schools: Skills Training for Emotional Problem Solving for Adolescents
PHLMS: Philadelphia Mindfulness Scale
SEL: social and emotional learning

## Data Availability

The data generated during this study are available from the corresponding author upon reasonable request.
